# Simultaneous Estimation of Hydrochlorothiazide, Hydralazine Hydrochloride, and Reserpine Using PCA, NAS, and NAS-PCA

**DOI:** 10.3797/scipharm.1505-03

**Published:** 2015-06-22

**Authors:** Chetan Sharma, Pragya Nand Badyal, Ravindra K. Rawal

**Affiliations:** Department of Pharmaceutical Analysis, Indo-Soviet Friendship College of Pharmacy (ISFCP), Moga 142001, India

**Keywords:** Net Analyte Signal, Principal Component Analysis, Relative Standard Error of Prediction

## Abstract

In this study, new and feasible UV-visible spectrophotometric and multivariate spectrophotometric methods were described for the simultaneous determination of hydrochlorothiazide (HCTZ), hydralazine hydrochloride (H.HCl), and reserpine (RES) in combined pharmaceutical tablets. Methanol was used as a solvent for analysis and the whole UV region was scanned from 200–400 nm. The resolution was obtained by using multivariate methods such as the net analyte signal method (NAS), principal component analysis (PCA), and net analyte signal-principal component analysis (NAS-PCA) applied to the UV spectra of the mixture. The results obtained from all of the three methods were compared. NAS-PCA showed a lot of resolved data as compared to NAS and PCA. Thus, the NAS-PCA technique is a combination of NAS and PCA methods which is advantageous to obtain the information from overlapping results.

## Introduction

Hydrochlorothiazide, hydralazine hydrochloride, and reserpine are antihypertensive drugs used to lower blood pressure. Hydrochlorothiazide, 6-Chloro-1,1-dioxo-1,2,3,4-tetrahydro-1λ^6^,2,4-benzothiadiazine-7-sulfonamide, is a thiazide diuretic which reduces the blood volume by inhibiting the Na^+^Cl^−^ symport followed by a reduction in the reabsorption of Na^+^ [1].

Hydralazine hydrochloride, or 1-hydrazinylphthalazine hydrochloride, is also a diuretic drug belonging to the hydrazinophthalazine class. It is a directly-acting smooth muscle relaxant which acts as a vasodilator in arteries and arterioles. It decreases the peripheral resistance, which thereby lowers the blood pressure and decreases the afterload [2].

Reserpine, methyl (3β,16β,17α,18β,20α)-11,17-dimethoxy-18-[(3,4,5-trimethoxybenzoyl)oxy]yohimban-16-carboxylate, is an antihypertensive and antipsychotic drug obtained as an indole alkaloid from the roots of the plant *Rauwolfia serpentina*. It irreversibly blocks the vesicular monoamine transporter (VMAT) which is responsible for transportation of norepinephrine, serotonin, and dopamine in presynaptic nerve terminals. Reserpine blocks the transportation of these neurotransmitters and controls the level of high blood pressure [3].

**Fig. 1:**
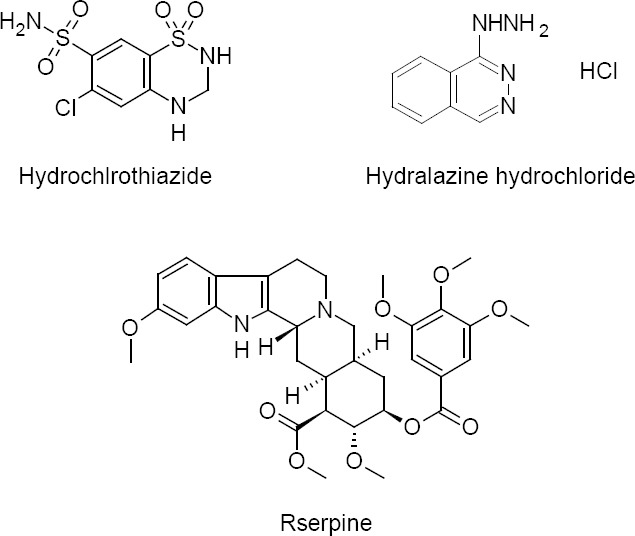
Chemical structure of hydrochlorothiazide, hydralazine hydrochloride, and reserpine

Very few analytical methods have been available for the quantitative and qualitative analysis of hydrochlorothiazide, hydralazine hydrochloride, and reserpine in formulation either individually or in combination with other drugs. Reserpine in antihypertensive tablets has been assayed by Fluorimetry and colorimetrically by using the 4-carboxyl-2,6-dintrobenzene diazonium ion [4, 5].

Simultaneous estimation of RES and HCTZ in tablet dosage form was performed by high-performance liquid chromatography (HPLC) using polythiazide as internal standard [6]. Simultaneous estimation of HCTZ and H.HCl was done by HPLC using dibutylamine phosphate buffer and ACN as mobile phase [7]. There is a variety of methods used for the estimation of HCTZ, H.HCl, and RES individually or in combination with other antihypertensive drugs using UV spectroscopy, HPLC, fluorescence spectroscopy, amperometry, and the time-resolved chemiluminescense method, but no method is available for the simultaneous estimation of all three drugs by UV spectroscopy and HPLC or any other method [8–12].

Therefore, our objective was to develop a new, rapid, and feasible simultaneous analytical UV/Vis spectrophotometric method combined with a multivariate calibration technique for the evaluation of HCTZ, H.HCl, and RES containing bulk drugs and combined tablet dosage forms because simultaneous estimation is less laborious, less time-consuming, and uses only a single solvent for analysis, which also reduces the cost of chemicals.

## Materials and Methods

### PCA Methodology

Principal component analysis (PCA) reduces the dimensionality of data sets comprising a large number of consistent variables. It explains the pattern of an inherent property of the individual objects. The main aim of the principal component analysis is the eigenvector decomposition of the covariance matrix of process variables [13, 14]. The covariance matrix for the data matrix (X) containing rows (m) and columns (n) is measured by equation 1:


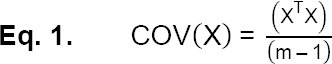


Principal components can be explained by equation 1.

General steps used in the PCA technique are as follows:

1. Subtraction of the mean
2. Calculation of the covariance by using equation (1)
3. Calculation of eigenvectors and eigenvalues of the covariance matrix
4. Dimensionality reduction and formation of the feature vector
5. Deriving the new data (final data)

### NAS Methodology

The net analyte signal (NAS) is a fraction of the spectral mixture which is directly correlated with the analyte concentration [15]. NAS is orthogonal to the spectrum of all components present in the sample except the analyte to be determined. Different steps that occur in the NAS methodology are:

- Determination of no. of analytes (p)
- Preparation of mixture standard solutions (j)
- Recording the absorbance spectra of the solutions at the (i) sensors (R matrix)
- Recording the absorbance spectrum of the unknown (r_un_ vector)
- Calculation of R_-k_

The NAS calculation has been performed in different steps:

1. NAS calculation for the calibration set:
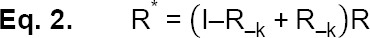
2. NAS calculation for the unknown sample:
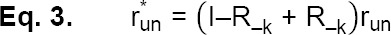
3. Calculation of pure NAS:
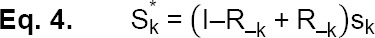

where R is the matrix of the mixture spectra

r_un_ is the matrix of the unknown sample

R_-k_ is the matrix of the background (other analytes + interferences)

### NAS-PCA Methodology

The net analyte signal-principal component analysis (NAS-PCA) is a combination tool of NAS and PCA which helps in the simultaneous estimation of pharmaceuticals. Statistical analysis has been performed by using equation 5:


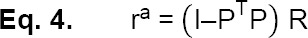


where P is the projection matrix generated on the orthogonal basis spectra composed of the principal components of R_-a._

The first few components of PCA were computed by using the non-linear iterative partial least squares (NIPALS) algorithm. Then the standard addition graph was plotted between r^a^ and added concentration c.

## Experimental

### Chemicals and Reagents

The reference standards of hydrochlorothiazide (HCTZ), hydralazine hydrochloride (H.HCl), and reserpine (RES) were supplied as gift samples by Dwarkesh Pharmaceutical Pvt. Ltd., Ahmadabad, Chimak Healthcare Solan (H.P.) and Rajasthan Antibiotics Ltd., New Delhi (India), respectively. All the working solutions for the analytical determination were prepared in analytical grade methanol which was purchased from Loba Chemie. The pharmaceutical formulations (tablet) of HCTZ, H.HCl, and RES were purchased from the market (Tab. SER-AP-ES^®^).

### Instrumentation and Software

The entire UV-Vis spectrophotometric measurements were made with a Perkin Elmer UV-Visible Spectrophotometer (Lambda 35) with a fixed slit width of 1 nm operated by Perkin Elmer UV Probe software version 2.31. The complete spectra were saved in CSV (Excel file) format and then the data were statistically analysed by using Unscrambler^®^ 10.3.0.89 software.

### Preparation of Standard Solutions

#### Standard Stock Solutions

HCTZ, H.HCl, and RES reference standards (10 mg) were accurately weighed and transferred to 10 mL-volumetric flasks separately. They were dissolved and diluted to 10 mL with methanol to obtain a stock solution of HCTZ, H.HCl, and RES with a final concentration of 1 mg/mL (1000 μg/mL).

#### Working Standard Solutions

Aliquots (1 mL) of standard solution of HCTZ, H.HCl, and RES were pipetted out and transferred to 10 mL-volumetric flasks separately and diluted to 10 mL with methanol to obtain working standard solutions of HCTZ, H.HCl, and RES with a final concentration of 100 μg/mL.

### Calibration Curve for the UV Method

**Fig. 2:**
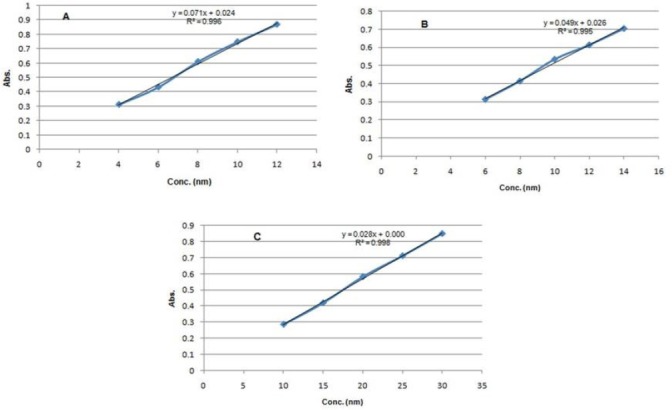
Standard plot of (A) HCTZ, (B) H.HCl, and (C) RES in methanol

Suitable amounts of aliquots of HCTZ, H.HCl, and RES were pipetted out and transferred into a series of 10 mL-volumetric flasks. The volume was made up to the mark with methanol to get a concentration of 4–12 µg/mL for HCTZ, 6–14 µg/mL for H.HCl, and 10–30 µg/mL for RES, respectively. All the samples were measured for their absorbances in the UV-Vis spectrophotometer using methanol as a blank. The standard plot was plotted for HCTZ, H.HCl, and RES which is given in Figure 2.

### Calibration and Validation Set for Analytical Estimation

For calibration purposes, a training set of 16 mixtures was prepared by mixing an appropriate volume of standard dilutions for 16 different concentration levels in individual 10 mL-volumetric flasks (Table 1). For validation purposes, a set of 10 synthetic ternary mixtures were prepared for which measurement was done in six replicates at each time for evaluating inter- and intraday variations. The UV absorption spectra wererecorded over the wavelength range of 200–400 nm. A total of 201 data points were obtained in each and every multivariate tool at an interval of 1 nm. The absorbance data of the calibration set obtained from the UV spectra were exported into an Excel file and then subjected to the Unscrambler® program for the NAS, PCA, and NAS-PCA tools.

**Tab. 1:**
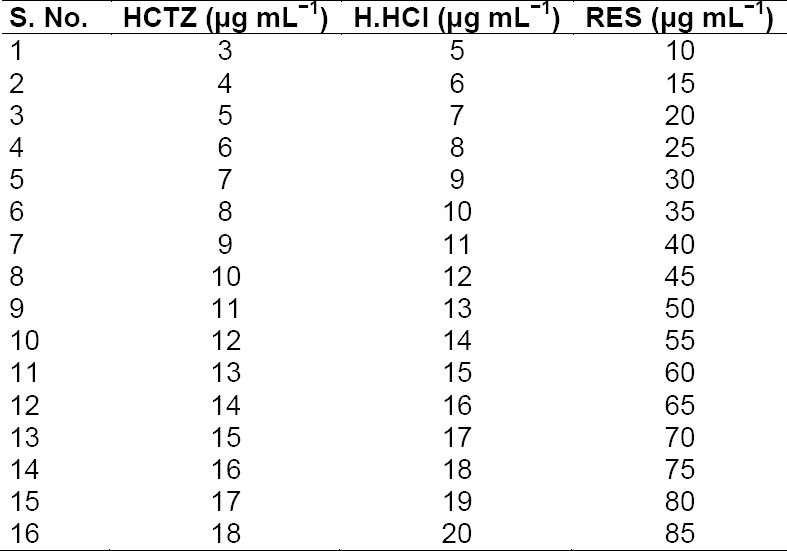
Composition of the calibration set

### Analysis of the Marketed Formulation

The average weight of 10 tablets was calculated and the tablets were finely powdered. Powder equivalent to one tablet was weighed properly and transferred into a 10 mL-volumetric flask. An amount of 5–6 mL methanol was added to the volumetric flask followed by sonication for 10 minutes. After sonication, the volume was made up with methanol and the solution was filtered through Whatmann filter paper. The filtered solution was measured for its absorbance in the UV-Vis spectrophotometer.

## Results and Discussion

### Analysis of the Combination Mixtures and Pharmaceutical Preparations

**Fig. 3:**
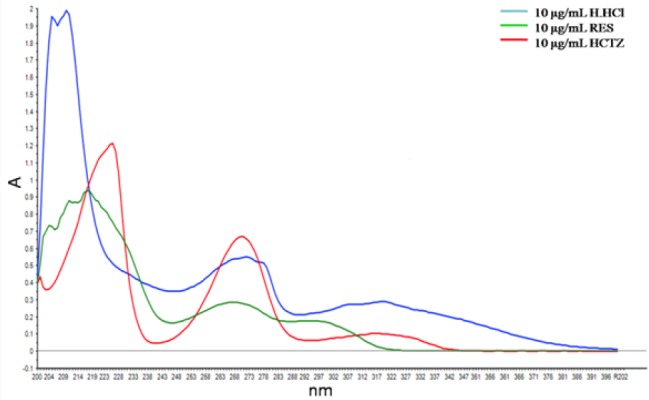
UV overlay spectrum of HCTZ (10 µg/mL), H.HCl (10 µg/mL), and RES (10 µg/mL) in methanol

A ternary mixture of HCTZ, H.HCl, and RES was scanned by a UV-Vis spectrophotometer in the range of 200–400 nm at an interval of 1 nm. The overlay spectra of these three drugs is shown in Fig. 3.

**Fig. 4:**
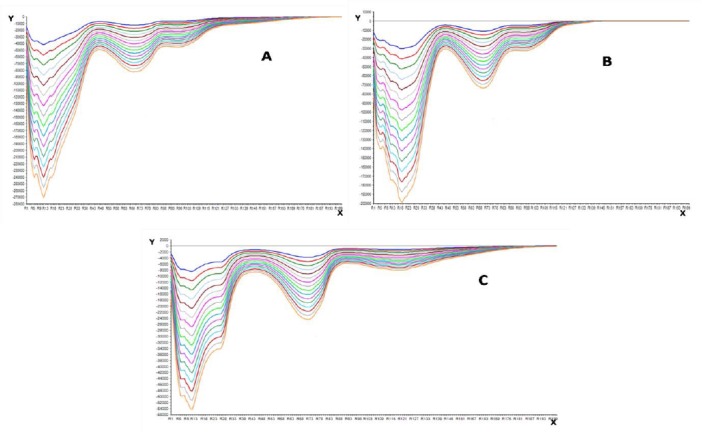
NAS curves obtained for (a) hydrochlorothiazide, (b) hydralazine hydrochloride, and (c) reserpine

**Fig. 5:**
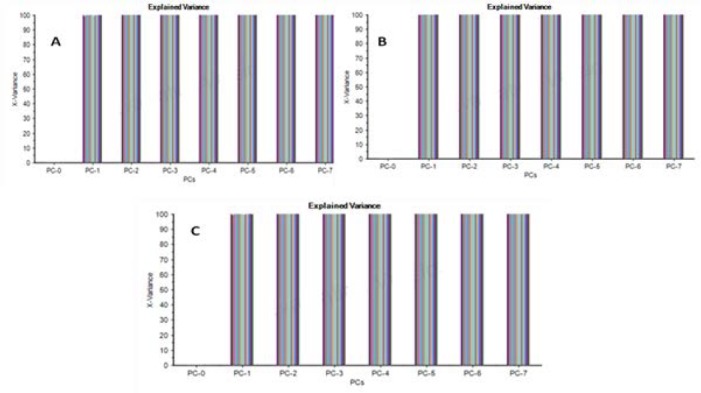
PCA curves obtained for (a) hydrochlorothiazide, (b) hydralazine hydrochloride, and (c) reserpine

**Fig. 6:**
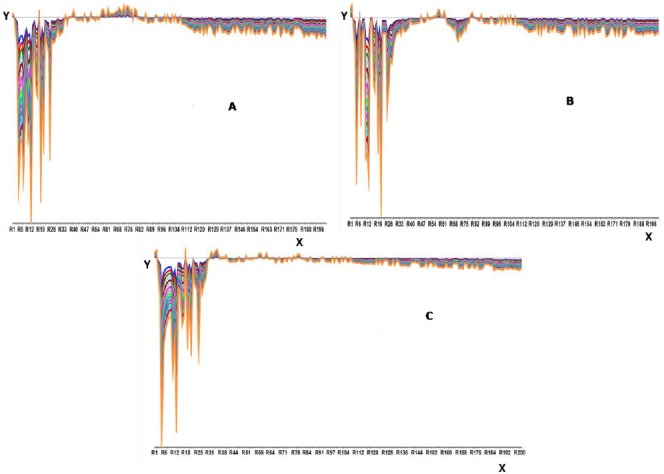
NAS-PCA curves obtained for (a) hydrochlorothiazide, (b) hydralazine hydrochloride, and (c) reserpine

Three multivariate tools, i.e. NAS, PCA, and NAS-PCA were used to resolve the data of the ternary mixtures of HCTZ, H.HCl, and RES. The data obtained was resolved by using NAS, PCA, and NAS-PCA for which curves of HCTZ, H.HCl, and RES are shown in Figs. 4–6, respectively.

The NAS-PCA tool gave better results as compared to PCA and NAS. The prediction error for every method in the mixture was calculated as the RSEP shown in Eq. 6:


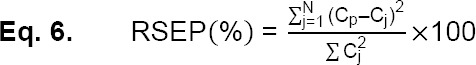


where *N* is the number of samples.

C_j_ and C_p_ are the concentrations of the components in the _jth_ mixture.

NAS-PCA gives higher percentage recovery and lower percentage RSEP as compared to NAS and PCA. The percentage recovery and percentage RSEP for the mixture sets were calculated and results are showed in Table 2.

**Tab. 2:**
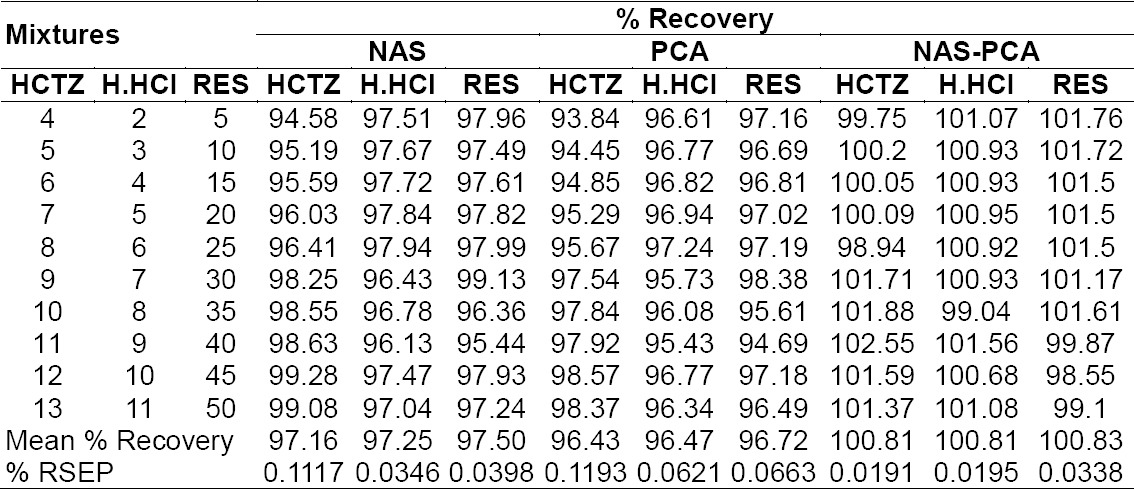
Composition and results of the analysis of the prepared mixtures by NAS, PCA, and NAS-PCA

All the values obtained from the NAS, PCA, and NAS-PCA methods were under the ICH limits. Analytical figures of merit and r^2^ values are given in Table 3. The marketed formulations obtained from the local market have been analysed (Table 4). NAS-PCA showed approximately 100% recovery for the marketed samples while in the case of PCA and NAS, it was approx. 98%, which is less than NAS-PCA.

**Tab. 3:**
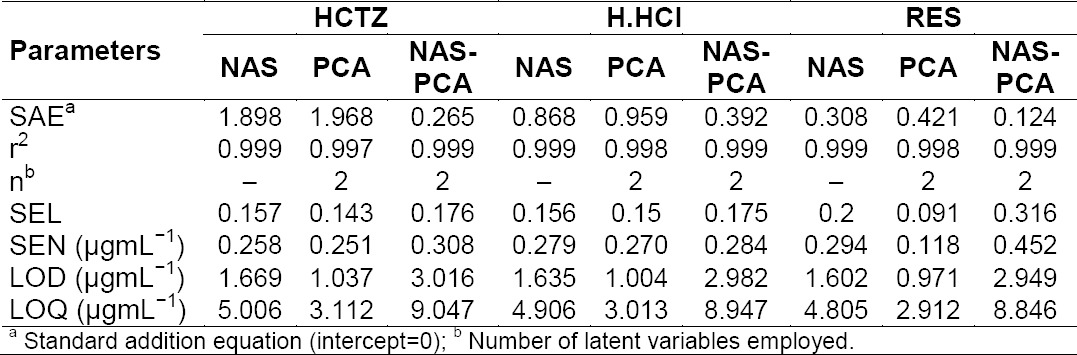
Composition and results of the analysis of data of prepared mixtures by NAS, PCA, and NAS-PCA

**Tab. 4:**
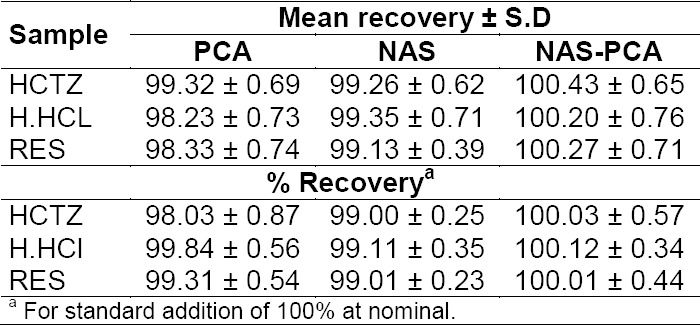
Determination of HCTZ, H.HCl, and RES in pharmaceuticals using the proposed methods

**Tab. 5:**
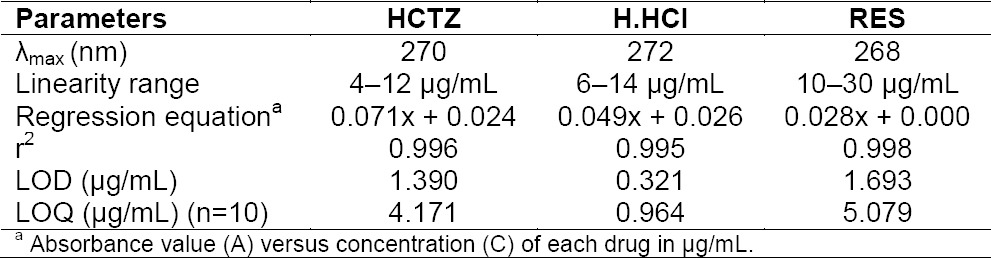
Analytical data from the calibration graphs for the determination of HCTZ, H.HCl, and RES by spectrophotometry

## Conclusion

All three methods were found to be very accurate, precise, and have good recoveries. The results obtained from the NAS-PCA method gave better results as compared to the NAS and PCA methods individually. The percentage RSEP value of NAS-PCA was lower with a good detection limit as compared to NAS and PCA. These multivariate tools are time-saving, less laborious, and inexpensive compared to other analytical techniques, i.e. HPLC, HPTLC, GC, LC-MS, GC-MS, etc. With an increasing number of interferences, the NAS-PCA signals did not have any effect on their results. It was found to be a robust tool. Multivariate tools adopted for the simultaneous estimation confirm to be acceptable to the label claim and signify the high accuracy and precision of the proposed method when applied to tablets.
